# Medical Education in Europe

**Published:** 1912-08-15

**Authors:** 


### MEDICAL EDUCATION IN EUROPE.

The Carnegie Foundation for the Advancement of Teaching has just issued its second bulletin on medical education[1], this time dealing with conditions found in Europe. This report, similar to the one issued two years ago, is based on investigations made by Mr. Abraham Flexner for the foundation. The report confirms the statements made by the Council on Medical Education four years ago[2], that the medical schools in this country are backward not only in the matter of preliminary education but also in the length of the medical course. It also supports the Council's statement that this is the only civilized country which does not require of every medical student a preliminary education in physics, chemistry and biology either prior to or at the beginning of the medical course.

In the introduction to the report, President Pritchett emphasizes several facts which are of extreme importance. In the first place no other country was found to have con-

1. Flexner, Abraham: Medical Education in Europe, Bull. No. 6, Carnegie Foundation, 576 Fifth Avenue, New York.

2. American Medical Association Bulletin, May 15, 1908, p. 234; The Journal A. M. A., Aug. 20, 1910, p. 680.

ditions so bad as those found in the United States, or as President Pritchett expresses it, "scandals in medical education exist in America alone." Again, "no medical school that lacks proper facilities has any other motive than the selfish advantage of those that carry it on; and no civilized country except America at this day allows such enterprises to impose on the public." Another point which is distinctly brought out in this report is the close relationship existing between the hospitals of Europe and the medical schools—a marked contrast to conditions in America. Well-trained young physicians in Germany find no difficulty in attaching themselves to the retinue of hospital staff physicians and surgeons and thus procuring for themselves active, scientific work, while in America this is practically impossible. In support of a statement so frequently repeated of late by those striving for higher educational standards in this country, President Pritchett says that no hospital can suffer by giving teaching privileges to a rightly conducted university medical school. The report shows that in Europe the trustees of hospitals freely open their wards to students and that the hospitals realize that such relationship is advantageous not only to medical science but also to the patients in the hospitals.

The report again furnishes a definite reply to the worn-out argument that cheap schools are needed to furnish doctors for sparsely settled districts of the country. It is stated that no physician, whether he be poorly equipped or well equipped will seek a location where a livelihood cannot be gained. On the other hand, it was shown that well-educated physicians will often prefer the sparsely settled districts if the means of a livelihood are to be obtained there. In other words, the experience of Germany shows that the distribution of physicians does not depend on a low standard of education. The report brings out the fact also that sectarian medicine is practically unknown in Europe and that this is the only country in which educationa institutions professing to teach sectarian medicine are to be found.

President Pritchett concludes his introduction to the report with the plea not only for the giving of larger sums to medical education in this country but also for the use of a proper discrimination in such giving. He shows that the problem in America will be more quickly solved if donations are given to the medical schools which deserve to exist and not to the undeserving. In contrast with conditions in this country, it is shown that in Germany all medical schools are supported by the government, with the result that in that country medical training, medical laboratories and teaching facilities in all the medical schools are on a uniformly high plane.

The report has apparently been widely circulated and already much attention has been given to it by the newspapers of the country. This is as it should be. Although there are those who deplore the publicity being given to the shortcomings of medical education in this country, nevertheless it is that very publicity which will the sooner bring about a removal of the disgrace and the establishment of better conditions.

This report on medical education abroad comes at a very opportune time. We are already in the midst of a rapid development of medical education and this report, based on the investigation of European medical schools, made at first hand, will be a great help in the solution of many of our problems. Although, by contrast with medical education abroad, many defects are seen in conditions in this country, the many improvements already brought about leave no ground for discouragement. It must not be forgotten that although the determined movement for better medical education in this country began only about eight years ago, astonishing progress has been made since that time. Our vast oversupply of inferior medical schools is rapidly being reduced. Where there were 166 medical schools in the United States in 1904—nearly half of the world's supply—now there are only 118. On the other hand, the number of colleges which have high standards for admission and which are equipped to do good work is rapidly increasing. Instead of only three in 1904 there are now forty-six which are requiring one or more years of collegiate work for admission. Again, state licensing boards are rapidly improving their standards and strengthening their examinations, which is helping to solve the problem. From present indications, therefore, it will need only a few more years to bring the majority of our medical schools both in their requirements of preliminary education and in the quality of their teaching up to a point that will compare quite favorably with the standards upheld in other civilized countries.—*Journal A. M. A.*, July 6, 1912.

